# Clinical and pathological characteristics of cervical clear cell carcinoma in patients not exposed to diethylstilbestrol: a comprehensive analysis of 49 cases

**DOI:** 10.3389/fonc.2024.1430742

**Published:** 2024-07-11

**Authors:** Jing Zeng, Wei Jiang, Kemin Li, Mengpei Zhang, Jinghong Chen, Yuanqiong Duan, Qingli Li, Rutie Yin

**Affiliations:** ^1^ Department of Obstetrics and Gynecology, West China Second University Hospital, Sichuan University, Chengdu, China; ^2^ Key Laboratory of Birth Defects and Related Diseases of Women and Children, West China Second University Hospital, Sichuan University, Chengdu, China; ^3^ Department of Pathology, West China Second University Hospital, Sichuan University, Chengdu, China

**Keywords:** adjuvant chemotherapy, adjuvant radiotherapy, cervical clear cell adenocarcinoma, fertility preservation, prognosis

## Abstract

**Purpose:**

This study aimed to investigate the clinical and pathological characteristics, treatment strategies, and prognosis of cervical clear cell carcinoma (CCCC) in patients not exposed to diethylstilbestrol *in utero*

**Methods:**

The patients diagnosed with CCCC at West China Second University Hospital of Sichuan University between January 2011 and Jun 2023 were enrolled for this retrospective study. The clinical characteristics and information on treatment and follow-up were collected. The Kaplan–Meier method and Cox regression analysis were performed to identify the relative variables for predicting progression-free survival (PFS) and overall survival (OS).

**Results:**

Of the 49 patients included, the Federation International of Gynecology and Obstetrics (FIGO) (2018) stage distribution was 37 (75.5%) stage I, 6 (12.2%) stage II, and 6 (12.2%) stage III. The median follow-up interval was 24.1 months. Six (12.2%) patients had a recurrence, and five (10.2%) patients died. The 5-year PFS rate was 86.8%, and the 5-year OS rate was 88.2%. No recurrence or death was detected in two patients who successfully completed fertility-preserving treatment and seven patients who underwent surgery to preserve ovaries. Two patients became pregnant, giving birth to two babies. The univariate analysis showed that FIGO stage, Pelvic lymph node (PLN) metastasis, lymph vascular space invasion, and depth of stromal invasion (*P* < 0.05) were significantly associated with PFS and OS. However, no significant prognostic factors were identified in the multivariate analysis.

**Conclusion:**

Ovary-preserving treatment and fertility-preserving surgery are safe and feasible in early-stage CCCC. Surveillance other than adjuvant treatment may be a better choice for early-stage CCCC without any pathological risk factors. More targeted therapies and immunotherapy should be pursued in future studies.

## Introduction

The etiology of cervical cancer is firmly linked to high-risk human papillomavirus (HPV) infection; however, only 60%–100% of cervical adenocarcinomas (ADCs) are associated with high-risk HPV (hrHPV) infection (1–3). In 2020, the World Health Organization (WHO) reclassified cervical ADCs into HPV-associated (HPVA) adenocarcinomas and HPV-independent (HPVI) ADCs according to their etiologic link to HPV infection, as well as morphology (4, 5). HPVI ADCs include gastric, clear cell, endometrioid, miscellaneous, and not otherwise specified types (4), which have type-specific pathogenesis, clinicopathological characteristics, and prognosis.

Cervical clear cell carcinoma (CCCC) is a rare subtype of ADC, accounting for only 4%–9% of cervical ADC (6). In 1971, Herbst reported a significant correlation between CCCC and *in utero* exposure to diethylstilbestrol (DES) (7). The clinical characteristics and prognosis of DES-associated CCCC received extensive attention, and the long-term survival data were reported (8). However, CCCC has still been reported in some patients with no exposure to DES in recent years. The clinicopathological characteristics and prognostic factors of CCCC without documentation of DES exposure are still lacking. Currently, gynecologic oncologists use the therapy and treatment principle referring to that of cervical squamous cell carcinoma and usual-type cervical ADC.

Therefore, our study summarized the clinicopathological characteristics and prognostic factors of 49 patients with CCCC in the gynecologic cancer center in Southwest China from January 2011 to June 2023. Meanwhile, the survival and obstetrical outcomes of ovarian and fertility preservation in young patients were analyzed and reported. The findings of this study might contribute to clinical decision-making and serve as a useful supplement to existing knowledge of this rare disease.

## Materials and methods

### Study population and pathological review

**Figure 1:**
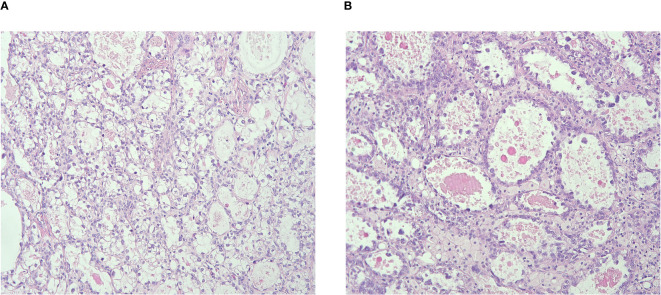
**(A)** Tumor cells presenting with abundant clear, glycogen-rich cytoplasm; **(B)** Tubulocystic structure with cuboidal, flattened, hobnail-shaped cells.

We identified 52 consecutive patients diagnosed with CCCC in West China Second University Hospital of Sichuan University between January 2011 and Jun 2023. Two senior pathologists reviewed the specimens from each patient, and three patients were excluded due to spread of clear cell ADC of the endometrium. All cases were diagnosed according to the WHO 2020 guidelines derived from the international endocervical ADC criteria and classification system (4, 5). The histological features of CCCC include three architectural patterns: solid, papillary, and tubulocystic structures, characterized by cuboidal, flattened, hobnail-shaped cells with abundant clear, glycogen-rich cytoplasm (**Figure 1**). The immunohistochemical markers are listed in **Table 1**.

**Table 1:** Immunohistochemical Markers of cervical clear cell adenocarcinoma.

Immunohistochemical markers	Percentage of positivity(%)
Napsin A	26/35(74.3)
HNF1β	26/27(96.3)
PAX8	36/36(100.0)
WT1	3/19(15.8)
ER	12/38(31.6)
PR	3/27(8.1)
P53,wide type	28/32(87.5)
Ki-67(>40%)	28/42(66.7)
CK7	30/30(100.0)
CK20	1/20(5.0)
CEA	2/13(15.4)
P16	33/40(82.5)

The data on demographics, clinical features, surgeries, pathologies, and adjuvant treatments were retrieved from individual medical records of the patients using the electronic medical record system. The stage of each patient was revised using the FIGO staging system of cervical cancer (2018) for consistency in statistical analysis (9). The follow-up information was obtained from the outpatient medical record system or via telephone call. Progression-free survival (PFS) was defined as the time of diagnosis until the date of clinically confirmed recurrence or death from any cause. Overall survival (OS) was calculated from the date of diagnosis to the date of death from any cause or the last follow-up date.

### Statistical analysis

The Kaplan–Meier method was used to estimate the hazard ratios related to PFS and OS. A multiple regression analysis was performed using the Cox proportional-hazards model to identify the relative importance of variables as factors for predicting PFS and OS. All statistical analyses were two sided, and statistical significance was considered when *P* was <0.05. The data were tabulated using Microsoft Excel software and analyzed using SPSS 20.0 software (IBM Inc., IL, USA).

### Ethics approval

This study was approved by the Ethics Committee of the West China Second University Hospital of Sichuan University. All procedures were performed following the guidelines and regulations of this ethics board. Informed consent was not required because of the retrospective nature of the study.

## Results

### Clinical features of CCCC

**Figure 2:**
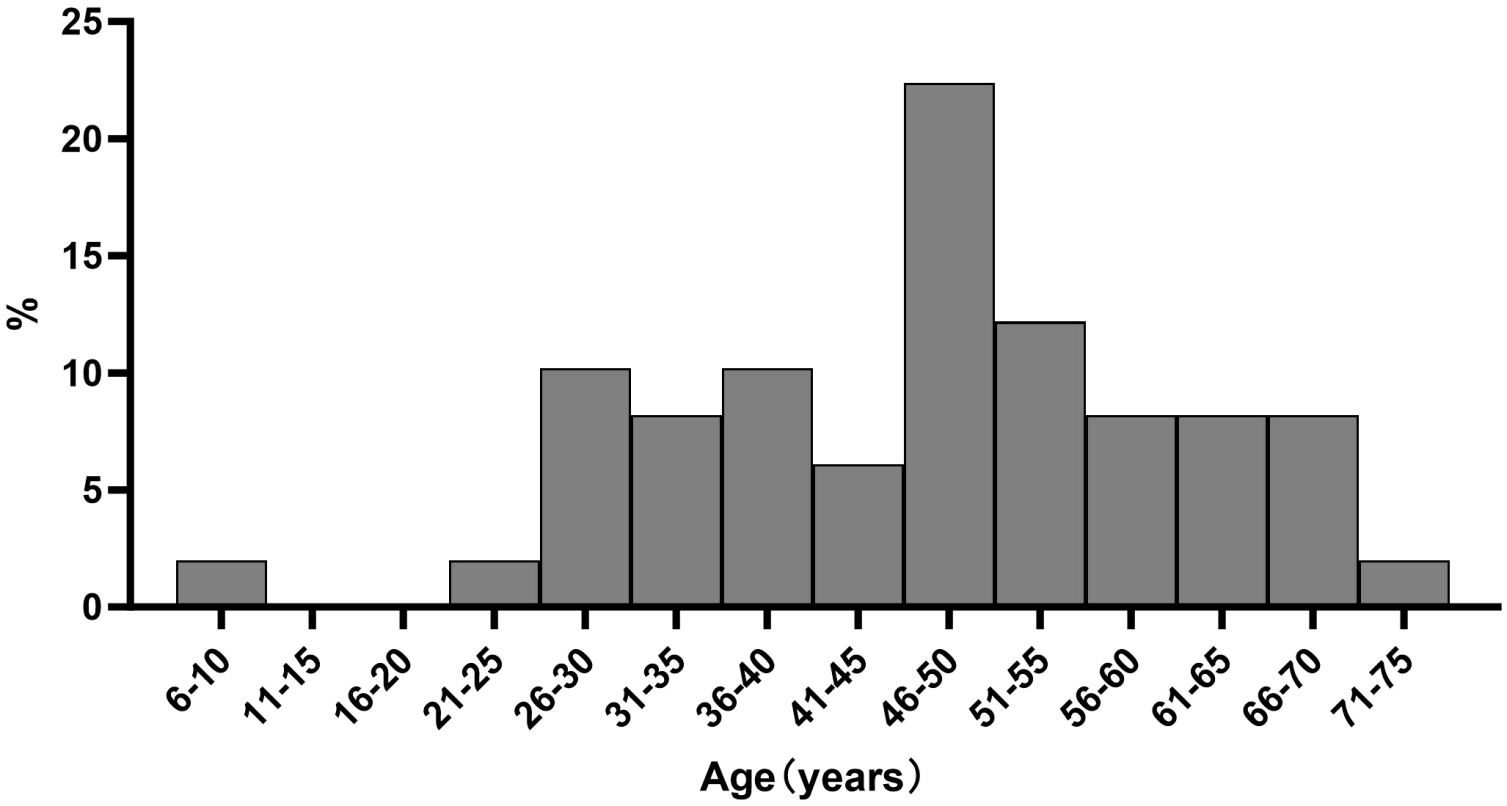
Age distribution of patients with cervical clear cell adenocarcinoma.

Forty-nine patients with CCCC were retrospectively analyzed. The median age at diagnosis was 48 years (range, 10–71 years). Further, 19 patients were aged less than or equal to 45 years (38.8%), and 30 patients were older than 45 years (61.2%). Seven patients were diagnosed before the age of 30 years. The age distribution is depicted in **Figure 2**. *In utero* DES exposure was documented in none of the patients. Thirty-six (73.5%) patients had the presenting symptoms of vaginal bleeding and four (8.2%) of vaginal discharge. Nine (18.3%) patients had no clinical symptoms, eight (16.3%) were diagnosed during a health check-up, and one (2.0%) was accidentally detected after a hysterectomy due to uterine myoma.

A total of 17 patients underwent cytological screening, and 27 HPV screening. Also, 6 (35.3%) of the 17 patients tested negative using the thinprep cytology test (TCT). Of the 11 (64.7%) patients with positive results, 2 (11.8%) had atypical squamous cells of undermined significance, 1 (5.9%) had a low-grade squamous intraepithelial lesion, 2 (11.8%) had a high-grade squamous intraepithelial lesion, 3 (17.6%) had atypical glandular cells, and 3 (17.6%) had ADC. Further, 19 (70.4%) patients tested negative using the HPV test. Among the remaining eight (39.6%) HPV-positive patients, the number of HPV16-positive patients was four (14.8%). Three (11.1%) patients had other high-risk HPV types, including HPV33, HPV51, and HPV52, and one (3.7%) patient had a low-risk HPV61 infection.

All patients underwent surgery. Forty-six patients underwent radical hysterectomy and pelvic lymphadenectomy. Among these, 41 patients underwent bilateral salpingo-oophorectomy, and 29 underwent para-aortic lymphadenectomy. Extrafacial hysterectomy was performed on one patient with a primary diagnosis of uterine myoma whose TCT and HPV screening was negative. Two patients underwent trachelectomy and pelvic lymphadenectomy. Further, 32 patients underwent laparotomy, and the remaining 17 patients underwent laparoscopy. The FIGO (2018) stage of the 49 patients was as follows: 18 (36.7%) stage IB1, 11 (22.4%) stage IB2, 8 (16.3%) stage IB3, 4 (8.2%) stage IIA1, 2 (4.1%) stage IIA2, 5 (10.2%) stage IIIC1P, and 1 (2.0%) stage IIIC2P. Lymph node metastasis was present in 6 (12.2%), absent in 41 (83.7%), and not reported in 2 (4.1%) patients. Vaginal cuff involvement was detected in 2 (4.1%) and deep cervical stromal invasion (more than one half) in 18 (36.7%) patients. Lymphovascular space invasion was present in 5 (10.2%) and absent in 44 (89.8%). Ovarian metastasis was present in two (2.0%) patients. None of the patients presented with parametrium or surgical margin involvement.

Among the 23 patients with risk factors (PLN metastasis, vaginal cuff involvement, tumor size more than 4 cm, lymph vascular space invasion, depth of stromal invasion, or metastasis to ovaries or fallopian tubes), 5 received adjuvant pelvic radiotherapy, 2 received consolidation chemotherapy, 13 received radiotherapy and chemotherapy, and 3 did not receive adjuvant therapy. Of 26 patients with no risk factors, 2 received adjuvant radiotherapy, 7 received consolidation chemotherapy, 7 received radiotherapy and chemotherapy, and 7 did not receive adjuvant therapy. Three patients (6.1%) had no data regarding adjuvant treatment. The external-beam radiotherapy (EBRT) dose was approximately 45–50 Gy with daily fractionation of 1.8–2.0 Gy. The brachytherapy fractionation schemes 6 Gy × 2 fractions dosed at 5 mm were used as a boost to EBRT in patients with positive vaginal mucosal surgical margins. Consolidation chemotherapy consisted of platinum-based regimens, including paclitaxel with cisplatin or paclitaxel with carboplatin for two to six cycles (**Table 2**).

**Table 2:** Clinicopathological characteristics of cervical clear cell adenocarcinoma.

Clinicopathological characteristics	CCCCs,n(%)
Age, median(range),y	48(10-71)
Age(y)	
≤45	19(38.8)
>45	30(61.2)
Presenting symptoms	
Vaginal bleeding	36(73.5)
Vaginal discharge	4(8.2)
Physical examination finding	8(16.3)
Accidental Found after BSO	1(2.0)
FIGO stage*	
I	37(75.5)
II	6(12.2)
III	6(12.2)
PLN metastasis	
No	41(83.7)
Yes	6(12.2)
NA	2(4.1)
Vaginal cuff involvement	
No	47(95.9)
Yes	2(4.1)
Tumor size,cm	
≤2	25(51.0)
>2, ≤4	13(26.5)
>4	11(22.4)
Lymph vascular space invasion	
No	44(89.8)
Yes	5(10.2)
Depth of stromal invasion	
Less than one half	31(63.3)
More than one half	18(36.7)
Metastasis in ovaries/fallopian tubes	
No	40(81.6)
Yes	2(2)
NA	7(14.3)
Surgery to preserve fertility	
No	47(95.9)
Yes	2(4.1)
Surgery to preserve ovaries	
No	42(85.7)
Yes	7(14.3)
surgical approaches	
Laparotomy	32(65.3)
Laparoscopy	17(36.7)
Adjuvant treatment	
No	10(20.4)
Single radiotherapy	7(14.3)
Single chemotherapy	9(18.4)
Radiotherapy and Chemotherapy	20(40.8)
NA	3(6.1)
Recurrences	
Yes	6(12.2)
No	43(87.8)
Recurrence site	
Lung	1(2.0)
Vaginal cuff	1(2.0)
Multisite involvement	1(2.0)
NA	3(6.1)
Death	
Yes	5(10.2)
No	44(89.8)

CCCC, cervical clear cell adenocarcinoma; BSO, Bilateral salpingo-oophorectomy; PLN, Pelvic lymph node; NA, not available.

*We use FIGO staging system: cervical carcinoma (FIGO 2018).

### Survival and obstetrical outcomes with fertility or ovarian function preservation

**Figure 3:**
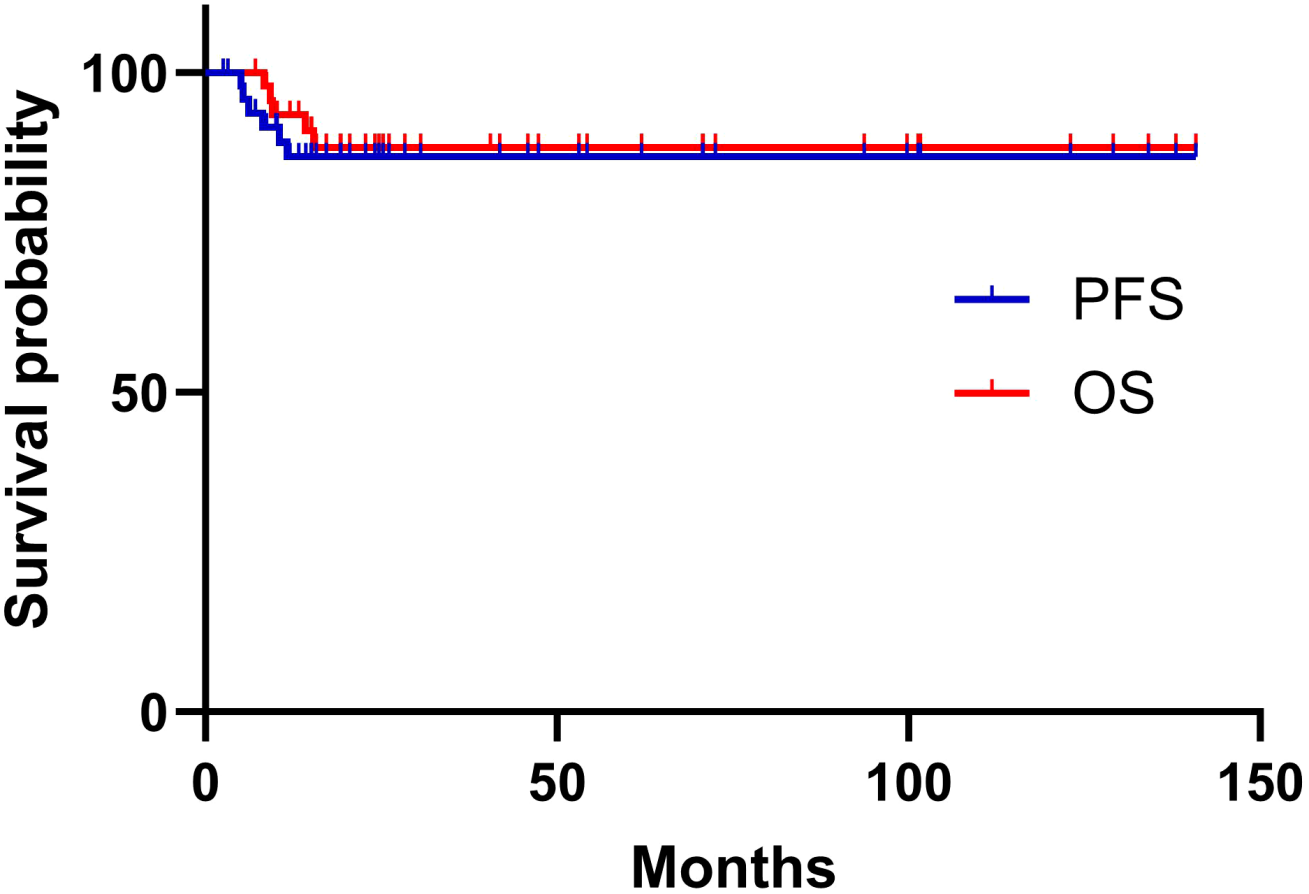
Kaplan-Meier survival curve for PFS and OS in 49 patients with CCCC.

The median follow-up interval was 24.1 months (range, 2.5–140 months). Six (12.2%) patients had recurrence. Sites of recurrence included lung (one patient), vaginal cuff (one patient), and multisite involvement (one patient); the information on site was not available for three patients. The 5-year PFS rate was 86.8%. The patient with lung metastases received chemotherapy, whereas the other five (10.2%) patients died of the disease. The 5-year OS rate was 88.2%. The survival curves are shown in **Figure 3**.

No recurrence or death was reported in two patients who successfully completed fertility-preserving treatment and seven patients who underwent ovary-preserving surgery. Two patients became pregnant, giving birth to two babies. Both patients conceived by *in vitro* fertilization–embryo transfer. One patient delivered at full term, and the other delivered at 26 + ^3^ weeks due to the premature rupture of membranes.

### Prognostic factors for CCCC

The univariate Cox regression analysis comparing clinicopathological parameters demonstrated that OS was influenced by FIGO stage (*P* = 0.001), PLN metastasis (*P* = 0.002), lymph vascular space invasion (*P* = 0.026), depth of stromal invasion (*P* = 0.025), and tumor size (*P* = 0.018). In contrast, PFS was influenced by FIGO stage (*P* = 0.000), PLN metastasis (*P* = 0.000), lymph vascular space invasion (*P* = 0.000), and depth of stromal invasion (*P* = 0.008). However, the multivariate analysis revealed no significant prognostic factors (**Table 3**).

**Table 3:** Univariate and multivariate survival analysis of associated clinicopathological characteristics with PFS in 49 cases.

Variables	Categories	PFS			OS		
		Univariate P Value	Multivariate P Value	Multivariate HR(95%CI)	Univariate P Value	Multivariate P Value	Multivariate HR(95%CI)
Age(y)	≤45	0.288			0.469		
	>45						
FIGO stage*	Stage I and II	0.000	0.106	0.81-8.78	0.001	0.133	0.75-8.40
	Stage III						
PLN metastasis	No	0.000			0.002		
	Yes						
Vaginal cuff involvement	No	0.131			0.633		
	Yes						
Tumor size, cm	≤2	0.077			0.018		
	>2						
Lymph vascular space invasion	No	0.000	0.746	0.16-13.50	0.026	0.868	0.07-9.09
	Yes						
Depth of stromal invasion	<1/2	0.008	0.252	0.36-46.70	0.025	0.249	0.37-48.08
	>1/2						
Metastasis in ovaries/fallopian tubes	No	0.559			0.583		
	Yes						
Surgery to preserve ovaries	No	0.340			0.392		
	Yes						
surgical approaches	Laparotomy	0.342			0.500		
	Laparoscopy						
Adjuvant treatment	No	0.941			0.835		
	Yes						

*FIGO stage: stage by The International Federation of Gynecology and Obstetrics.

## Discussion

Since 1971, Herbst et al. conducted a series of studies on clear cell carcinomas of the vagina and cervix in patients exposed to DES *in utero* (10). One of his studies reported that the risk of developing CCCC in a woman with no exposure to DES from birth through the age of 34 years was approximately 1 case per 1000 women (11). The use of DES during pregnancy was officially banned by the US Food and Drug Administration. The study also showed that the youngest patient exposed to DES who developed clear cell ADC was 7 years old at the time of diagnosis, whereas the oldest patient was 34 years (12). Further, 91% of DES-exposed women were diagnosed when they were between the ages of 15 and 27 years; the median age at diagnosis was 19 years (7). CCCC in patients not exposed to DES had bimodal age distributions, with the first peak among women aged 17–37 years (mean age 26 years) and the second among women aged 44–88 years (mean age 71 years) (6). Although the youngest patient in our study was only 10 years old, seven patients were less than 30 years old. However, the age distribution was not bimodal. CCCC occurred in all age groups, which was consistent with the findings of Seki et al. (13).

Although the WHO classification system (2020) reclassified CCCC as HPVI ADC, the association between CCCC and HPV remains controversial. Some studies found that the HPV infection rate in CCCC ranged from 0% to 33.3% (2, 14–16). The HPV infection rate in our study was 39.6%, which was slightly higher than the rate reported previously. Previous studies also reported that up to one third of patients with CCCC were P16 positive (4, 17). Therefore, the pathogenesis of CCCC in patients with no exposure to DES needs further exploration. The hit-and-run theory could explain the absence of the viral genome in HPVI ADCs. It proposed that once a viral infection caused sufficient cellular alteration, tumor maintenance no longer required the expression of viral proteins or viral infection, and thus the virus might be lost during cancer progression (18). The other reasons for undetected HPV infection were the presence of viral genotypes not included in the molecular tests and the failure in the detection of the diagnostic method employed. The occurrence of CCCC might also be related to *P53* gene mutation, Bcl-2 overexpression, instability of microsatellite repeats, and cervical endometriosis (19–21).

With the introduction and implementation of cervical cancer screening methods by the 1960s, the incidence of cervical cancer has decreased by 70% in the United States. At present, all countries in the world have established cervical cancer screening systems in line with their conditions. Also, HPV vaccination is gradually being rolled out in all countries, although the screening and early diagnosis of CCCC are still challenging. Pirog et al. provided comprehensive data on HPV genotype distribution in patients with different histological subtypes of tumors. They demonstrated that HPV testing and vaccination did not prevent CCCC (2). Previous studies reported an abnormal Pap smear of 18%–66.7% in patients with CCCC (22, 23). Our study revealed 64.7% abnormal TCT findings. The discrepancy in cervical cytology results was not only due to different pathologists but also related to the different cytologic techniques used. TCT was more effective in detecting cervical lesions than traditional smears. Tournaire et al. published CCCC screening recommendations for young women exposed to DES in France (24). The methods suggested the association of cytology and hrHPV testing with cervical and vaginal sampling. The screening timing was recommended as annual or not exceeding a 3-year interval, continuing after 65 years and after a hysterectomy. Effective screening methods for CCCC in patients without exposure to DES are still lacking. Therefore, timely cervical biopsy and endocervical curettage are essential in patients with positive cytology and hrHPV.

Thomas et al. conducted an outcome analysis of patients with CCCC after exposure to DES at three major gynecologic cancer centers between 1982 and 2004. The result showed that stage I or IIA CCCC was amenable to surgical resection and displayed an excellent 3-year OS of 91% (22). Hanselaar et al. reported similar survival rates for patients with early-stage CCCC (6). Therefore, some gynecologic oncologists continue to explore the use of fertility-preserving surgery for early-stage CCCC. However, cold-knife conization or radical trachelectomy has been recommended by the National Comprehensive Cancer Network guidelines for early-stage cervical cancer considering only the histological characteristics of squamous cell carcinoma, ADC, or adenosquamous carcinoma. Liu et al. reported six patients with stage IA-IB CCCC who successfully completed fertility-preserving treatment (16). No recurrence or death was reported in all patients, and two patients became pregnant with a live birth rate of 100%. In our study, two patients undergoing fertility-preserving surgery had stage IB CCCC with no other high-risk factors and remained disease free after 71 and 62 months, respectively. The pregnancy rate was 100%, and the live birth rate was 100%. It is safe and feasible for patients with early-stage CCCC to receive fertility-preserving treatment. Pelvic magnetic resonance imaging should be used to carefully evaluate the tumor size and the degree and extent of cervical canal invasion and also to exclude distant metastasis. Therefore, for patients with early-stage CCCC, a multidisciplinary team with cooperation is needed for comprehensive evaluation. Also, patients need to be informed that fertility-preserving surgery is not the standard treatment.

The use of ovary-preserving surgery for patients with non-squamous cell cervical carcinoma remains controversial. Many studies have reported an ovarian metastasis rate of 4.2%–16.3% in patients with non-squamous cell carcinoma (23, 25–27), which is 2.4%–4.3% in patients with CCCC (28, 29). In our study, the ovarian metastasis rate was 4.1%, which was similar to that in the aforementioned studies. No recurrence or death was reported in seven patients who underwent ovary-preserving surgery. In this study, the ovary-preserving treatment appeared safe and feasible in patients with early-stage CCCC. However, considering the limited sample size and the retrospective nature of our study, ovary-preserving surgery should still be cautiously selected for patients with CCCC.

The 5-year OS rate was 88.2% in our study, which was higher than previously reported survival rates of 40%–78% (4, 16, 22, 28–30). This was mainly because a high percentage of the study population comprised patients with early-stage CCCC who underwent surgery, and the other six patients with stage III CCCC had lymph node metastasis after surgery re-staged according to the 2018 FIGO staging. Previous studies reported that advanced stage and lymphatic involvement were associated with worse prognosis (16, 22, 28, 29). Thomas et al. found that patients with stage I or IIA CCCC had a better 3-year OS compared with patients with stage III or IV CCCC (91% vs 22%, *P* < 0.001), and the presence of positive lymph nodes had a negative impact on 5-year PFS (31% vs 92%, *P* = 0.001) and 5-year OS (80% vs 100%, *P* = 0.02) in patients with stages I and IIA CCCC (22). In our study, the univariate analysis showed that advanced stage (III), PLN metastasis, LVSI, and depth of stromal invasion were significantly associated with PFS and OS; however, the multivariate analysis revealed no significant prognostic factors. We also found that 26 patients in our population had no risk factors, 16 of which received adjuvant radiotherapy and/or chemotherapy and only 7 did not receive adjuvant therapy. However, adjuvant treatment did not affect survival outcomes in early-stage CCCC without any pathological risk factors. Surveillance other than adjuvant treatment might be a better choice for these patients.

In our study, six patients relapsed, of which 4 had stage III CCCC. Five patients received radiotherapy and chemotherapy, whereas one patient had no data regarding adjuvant treatment. Therefore, even adjuvant therapy did not seem to improve the prognosis of patients with high-risk prognostic factors. Five patients died, and only one patient who was alive underwent whole-exome sequencing, revealing suspected pathogenic mutations of AT-rich interaction domain 1A gene (*ARID1A*) and ataxia telangiectasia mutated (*ATM*) genes. She was treated with the mammalian target of rapamycin complex 1/2 (mTORC1/2) dual inhibitor ATG-008 combined with toripalimab after participating in one clinical trial, achieving a PFS of 9 months. Zorn et al. compared endometrial, ovarian, and renal clear cell carcinomas and reported that clear cell carcinomas had a remarkable similarity in gene expression profiles (31). This finding indicated the possibility of treating clear cell carcinomas in the same way, that is, some molecular events were the same irrespective of the organ of origin. Ueno et al. demonstrated increased epidermal growth factor receptor (EGFR) or human epidermal growth factor receptor-2 (HER2) expression or activation of protein kinase B (Akt) or mTOR in 13 patients with CCCC (15), indicating that the inhibitors of tyrosine kinases or the Akt–mTOR pathway might be suitable treatment regimens for CCCC. Therefore, we look forward to the clinical research data of targeted therapy and immune checkpoint inhibitors in CCCC.

### Limitations

Our study had certain limitations. First, the retrospective nature of the study led to inevitable missing data and bias. Second, the sample size of the study was small due to the low incidence of CCCC, and hence some subgroup analyses could not be performed. We hope to provide more evidence and data to improve the diagnosis and treatment of this rare disease by conducting international multicenter prospective studies in the future.

## Conclusions

CCCC is an HPVI-related cancer, and hence traditional screening methods are ineffective. Therefore, timely cervical biopsy and endocervical curettage are extremely important. Ovary- and fertility-preserving surgeries are safe and feasible due to the good prognosis of early-stage CCCC; however, we still recommend careful selection of treatment modalities because of the limited data on this issue. Chemotherapy and/or radiotherapy does not affect the survival of patients with early-stage cervical cancer. Therefore, more studies on targeted therapy and immunotherapy for CCCC should be conducted in the future.

## Data availability statement

The raw data supporting the conclusions of this article will be made available by the authors, without undue reservation.

## Ethics statement

The studies involving humans were approved by Medical Ethics Committee of West China Second University Hospital, Sichuan University. The studies were conducted in accordance with the local legislation and institutional requirements. The ethics committee/institutional review board waived the requirement of written informed consent for participation from the participants or the participants’ legal guardians/next of kin because of the retrospective nature of the study. And the human samples used in this study were acquired from electronic medical record system. Written informed consent for participation was not required from the participants or the participants’ legal guardians/next of kin in accordance with the national legislation and institutional requirements.

## Author contributions

JZ: Conceptualization, Data curation, Formal analysis, Funding acquisition, Investigation, Methodology, Project administration, Resources, Software, Writing – original draft, Writing – review & editing. WJ: Data curation, Resources, Visualization, Writing – original draft. KL: Data curation, Methodology, Writing – original draft. MZ: Data curation, Writing – original draft. JC: Data curation, Writing – original draft. YD: Data curation, Writing – original draft. QL: Conceptualization, Supervision, Validation, Writing – review & editing. RY: Conceptualization, Funding acquisition, Supervision, Validation, Writing – review & editing.
